# Detection of two Arctic birds in Greenland and an endangered bird in Korea using RGB and thermal cameras with an unmanned aerial vehicle (UAV)

**DOI:** 10.1371/journal.pone.0222088

**Published:** 2019-09-04

**Authors:** Won Young Lee, Mijin Park, Chang-Uk Hyun

**Affiliations:** 1 Division of Polar Life Sciences, Korea Polar Research Institute, Incheon, Republic of Korea; 2 Department of Forest Sciences, Seoul National University, Seoul, Republic of Korea; 3 Unit of Arctic Sea-Ice Prediction, Korea Polar Research Institute, Incheon, Republic of Korea; Wildlife Conservation Society Canada, CANADA

## Abstract

Unmanned aerial vehicles (UAVs), so-called ‘drones’, have been widely used to monitor wild animals. Here, we tested a UAV with red, green, and blue (RGB) and thermal cameras to detect free-living birds in a high Arctic region in North Greenland and in a restricted area in the Republic of Korea. Small flocks of molting pink-footed geese (*Anser brachyrhynchus*) near sea ice and incubating common ringed plovers (*Charadrius hiaticula*) in the Arctic environment were chosen for the RGB and thermal image studies. From the acquired images, we built mosaicked RGB images and coregistered thermal images, and estimated the animal shapes. Our results showed that geese were discriminated in both RGB and thermal images with water and sea ice backgrounds. Incubating plover bodies were not distinguished in RGB images due to their cryptic coloration, but they were detected in thermal images with cold background areas in the Arctic environment. We further conducted a blind survey in a restricted area under military control in Korea near the breeding sites of black-faced spoonbill (*Platalea minor*), which is an endangered species. From UAV flights with RGB and thermal cameras operated out of the restricted area, we acquired images of white objects in the mudflats and verified that the objects were resting spoonbills by watching the birds. We suggest that thermal cameras and UAVs can be applied to monitor animals in extreme environments and in restricted areas and help researchers find cryptic wader nests.

## Introduction

Recent technological advances in unmanned aerial vehicles (UAVs) have enabled exploration at fine spatial resolutions in many ecological studies. Using devices equipped with a UAV system, researchers study vegetation dynamics, ecosystem processes and animal population distributions at a large scale [1]. UAVs provide two main benefits: efficiency and effectiveness. From an efficiency perspective, UAVs are serviceable in reducing human bias, thus enhancing accuracy [2]. Massive data, such as a series of images, are obtained at a more reasonable price by UAVs than by manned aircrafts [3]. Manpower reduction is another benefit of UAVs; UAVs are useful for repetitive operations, especially when researchers have to conduct spatiotemporally consistent studies. Regarding their effectiveness, UAVs equipped with multiple sensors satisfy diverse purposes, such as the measurement of radiative heat fluxes over land and river [4] or topographical changes [5]. In addition to their efficiency and effectiveness, UAVs assure accessibility to dangerous environments and do not require equipment to be installed on the ground.

Emphasizing these strengths, UAVs are now widely used in wildlife research; in terms of habitat types, they are used from forests to polar regions, and with regard to taxa, mammals and birds are the predominant organisms recorded by UAV studies [6]. Early studies focused on testing the availability of UAVs for monitoring [3–9]. Prior to piloting UAVs in practical field application, a pattern recognition algorithm was developed to count birds using decoys [7]. A similar methodology was performed in the ocean with inflatable kayaks, representing whale-like targets [8]. Later, UAVs were preferentially applied to detect large, terrestrial animals and employed to investigate aquatic wildlife living in inland waters or the ocean. With recent technologies of high-resolution photography, researchers have adopted this device to monitor bird nesting at a distance, such as by counting the distribution of the black-headed gull (*Chroicocephalus ridibundus*) nests [9]. Moreover, by piloting UAVs above the canopy, Junda et al. [10] could ascertain all nest contents in the area and determine the species of the eggs belonged to, the clutch size, and the number of nestlings.

The application of UAVs in polar regions is intensely anticipated, especially in glaciology [11]. There have been limitations to monitoring in polar regions because of the lack of accessibility to sea ice, but the use of UAVs may cast light on this matter. Because UAVs can cover several square kilometers depending on their size [1] and aviate back to the operators, UAVs are well suited for investigating polar regions [12–14]. In particular, there are many geese molting on sea ice during the summer season. Because molting geese are not able to fly, they gather together and float near or on ice floes as an adaptation against the risk of predation. Thus, it is difficult for researchers to monitor them at a close distance due to their sensitivity during molt. Furthermore, many wader species also visit the Arctic for breeding. Most waders are cryptic when incubating nests. Waders have plumage with similar colors and patterns of their background [15]. Some species crouch motionlessly and protect their nests under predation risk [16]. Therefore, nesting birds are very well concealed, which hinders the location of wader nests from being determined by researchers.

UAVs have strengths for monitoring endangered species because these species sometimes prefer to live in uninhabited islands or restricted military zones to avoid human disturbance [17–19]. Thermal cameras have frequently been employed since the 1960s and have mainly been used to detect nocturnal behavior [20]. A recent lapwing (*Vanellus vanellus*) case study showed that a thermal camera and UAV can be applied to detect incubating nests [21]. We expect that a thermal camera can be used to find such geese on seawater and cryptic wader bird nests on the ground because this type of camera enables researchers to discriminate warm birds from their environments. In thermal images from the Arctic environment, a relatively high temperature body is easily distinguishable compared to the cold-temperature ground. Conversely, hot backgrounds can be discriminated from the lower radiation temperature of animals in temperate or tropical areas.

Here, we tested a UAV system with red, green, and blue (RGB) and thermal cameras in a high Arctic area and in a restricted area. In Northeast Greenland National Park, we tested a UAV detection technique on molting pink-footed geese (*Anser brachyrhynchus*) and incubating common ringed plover (*Charadrius hiaticula*). To best our knowledge, this is the first to apply UAV with RGB and thermal cameras for monitoring molting geese and breeding waders in the high Arctic. Additionally, for a blind survey in the restricted area of Incheon, the Republic of Korea, black-faced spoonbills (*Platalea minor*), an endangered species, was observed using our UAV flight method.

## Materials and methods

### Ethical statement

This research has been conducted under permissions from the Greenland government, and was granted a permit that includes the consideration and approval of monitoring pink-footed geese and common ringed plover with unmanned aerial vehicles (permission no. G16-074, C-17-4, C-18-4-09). The local ethics committee (Ministry of Industry and Mineral Resources, the Government of Greenland) specifically reviewed and approved the application for survey license on the use of biological resources for commercial and research purposes (in the following: Act on Biological Resources). Additionally, flights in the Republic of Korea were approbated according to the Division of Intelligence Information of the Republic of Korea Army (permission no. 1380). The decision of the exact study location was made in consultation with the Incheon Metropolitan City government and the local environmental organization to avoid disturbing the breeding sites of black-faced spoonbills. All applicable international, national, and/or institutional guidelines for the care and use of animals were followed.

### Study site and populations

**Fig 1:**
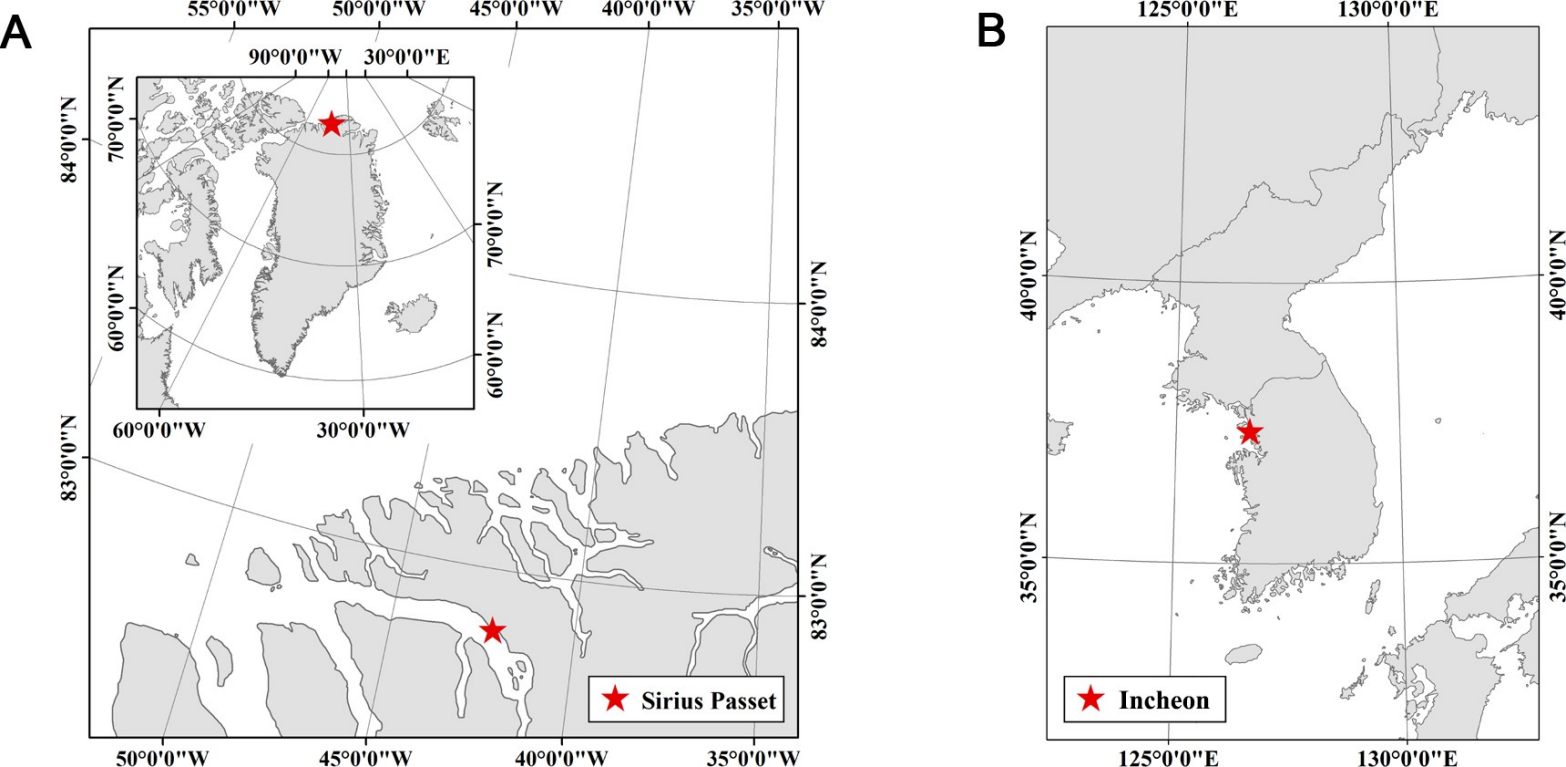
The location of our study site. (A) Sirius Passet, at the latitude 82°47.6´N and longitude 42°13.7´W in North Greenland. (B) Incheon, 37°21.1’N, 126°39.1’E in the Republic of Korea.

On 18 July 2018, we visited Sirius Passet on the eastern shore of J. P. Koch Fjord in North Greenland. This is a far north Arctic area, which is located at the latitude 82°47.6’N and longitude 42°13.7’W (Fig 1). There are large populations of molting pink-footed geese in this area [22, 23] and four wader species, including common ringed plover [23]. Since 2016, the same expedition team of six researchers has been annually visiting the study site and monitoring the breeding status of birds during the breeding season [23]. A daily census was conducted in a survey area of approximately 5 km^2^, and the bird nest positions were recorded with a GPS (Geo7x handheld, Trimble GeoExplorer, Sunnyvale, CA, USA; Global Navigation Satellite System (GNSS) accuracy of 1–100 cm). The number of eggs and hatching dates were recorded. GPS positions were used for mapping the nest sites in this area included in another study on the breeding survey, which had been continued since 2016 [23]. The survey have been performed before the flights. The geese were observed near the seashore when the birds were resting on the sea ice or floating on melted water. Small flocks were observed near the sea. The molting individuals were very sensitive to human approach, so we kept a distance of approximately over one hundred meter from the flocks in order to not disturb the birds. Common ringed plover nests were found near the streams with rocks. Four breeding nests were found during the field survey in 2018.

On 17 April 2019, we monitored a small mudflat area of 0.05 km^2^ along the Yellow Sea coast in Incheon, Republic of Korea. At latitude 37°21.1’N and longitude 126°39.1’E (Fig 1). This area is under landfill construction and restricted for military control purposes [24]. This area was chosen because birds are expected to feed near the mud flat, but no human approach is allowed due to military purposes. Additionally, there is a breeding site of black-faced spoonbills 5 km north of this area (“Songdo” district) [24]. Thus, we expected to observe spoonbills in the mud flat.

### Flight sites and conditions

We controlled a quadcopter drone (Phantom 4 advanced, DJI co.) over molting pink-footed geese and common ringed plovers incubating nests in Sirius Passet, North Greenland (Fig 1A). We approached three flocks of pink-footed geese once on the 18^th^ of July under sunny weather without strong wind. UAV flights were conducted near 4 pm (in local time) near the seashore for pink-footed geese. The air temperature was 7.8°C, and the relative humidity was 72%. One incubating common ringed plover was approached at 5 pm on 16 July. Similarly, the temperature was 8.0°C, and the relative humidity was 72%. The drone weighed 1,380 g, and its diagonal size excluding the propellers was 350 mm. This device had GPS and inertial measurement unit (IMU) functions to help autonomous flyers move along controlled routes during flights. The drone was equipped with a visible light (RGB) camera (Phantom 4 camera with 1-inch 20 MP sensor) and an additional external thermal camera (FLIR Vue Pro R, 13 mm lens, 640 × 512 pixels sensor) was mounted on the body of the UAV with nadir looking geometry.

Additionally, for a blind test, we operated two quadcopter drones on the 17^th^ of April, 2019 in Incheon, the Republic of Korea (Fig 1B), when the weather was sunny without strong wind. UAV flights were conducted at approximately 2 pm (in local time), approximately one hour ahead of the low tide. The air temperature was 17°C and the relative humidity was 49%. Due to the mechanical problems in attachment of a thermal camera to the drone with RGB camera, we had to perform two separate flights with two different vehicles: one with RGB camera and the other with the thermal camera. The first approach was conducted by a quadcopter drone (DAYA 550) weighing 1,500 g equipped with an external thermal camera (the same FLIR Vue Pro R that we used in Greenland). Then, after approximately 10 min, the second approach was performed by a quadcopter drone (Phantom 4, DJI co.) with an RGB camera (Phantom 4 camera with 1-inch 20 MP sensor).

### Image acquisition and processing

Boertmann et al. [16] previously conducted the monitoring of three geese species (pink-footed geese, Barnacle Goose *Branta leucopsis* and Light-bellied Brent Goose *Branta bernicla hrota*), but only pink-footed geese were counted in our study area (8517 individuals). Lee [23] performed conventional monitoring on land and observed pink-footed geese individuals in the molting stage. In this study, we also detected pink-footed geese flocks at the seashore by two observers during the daily survey. The molting individuals did not allow humans to approach closely and avoided the approaching humans by moving to the open water [23]. For the drone images of pink-footed geese, we chose three flocks at the seashore (16, 16, and 5 individuals), which had been previously detected by binoculars (Zeiss Victory FL, 10×42). Previous studies indicated that seabirds show species and status-specific behavioral response [14, 25, 26], but over 100 m flights were recommended [26] for approaching resting individual birds. Thus, we conducted UAV flights at 110 m above ground level (AGL) for 20 min, and the operator was on a hill approximately 500 m away from the birds.

The UAV moved at a speed of 5 m/s, and RGB images were manually taken by a remote control when birds were seen in the control screen. The thermal camera was automatically set to take images every second using the minimum interval of the thermal camera to collect as many thermal images as possible during the UAV flights within the limited fieldwork period. After the flight, we selected 24 RGB images and 69 thermal images, which covered the geese and the surrounding area; there was enough overlap among images that at least three images overlapped per pixel in the target area, preventing gaps between images. The RGB images were then mosaicked using structure from motion (SfM)-based PhotoScan Pro software (Agisoft LLC, St. Petersburg, Russia). The RGB image mosaicking was performed with the following procedure: (i) image alignment, (ii) sparse point detection, (iii) dense point cloud construction, (iv) digital elevation model (DEM) generation, and (v) image orthorectification and mosaicking (e.g., [21]). The ground sampling distance (GSD) values of the mosaicked RGB image and coregistered thermal image were 4.19 cm and 20.37 cm, respectively.

The thermal images were not mosaicked because of the temperature discrepancy between images caused by the thermal camera’s measurement accuracy, i.e., ± 5°C. Instead, a single thermal image showing the best sharpness was selected and coregistered to the higher resolution RGB mosaic image with carefully and manually selected tie points. A thermal image containing geese was used to compare the temperature of geese with the temperature of surrounding areas (ocean, sea ice and land). Using the ‘Create Random Points’ tool in ArcGIS 10.3 (ESRI, Redlands, CA, US), 120 random points were produced from a thermal image. Among the 120 points, 110 points were assigned to one of three surroundings (ocean, sea ice, land) and 10 points assigned to the boundaries were discarded.

In a previous study on nesting locations of Black-vented Shearwater (*Puffinus opisthomelas*), drones flew down to 25 m AGL, and no strong response was observed [25]. In other seabird studies on breeding nests, Gentoo (*Pygoscelis papua*) and Adélie penguins (*Pygoscelis adeliae*) were reported to exhibit strong responses to drones at low altitudes of 10–20 m in Antarctica [13], and 11 southern seabird species showed strong behavioral postures in response to drones at 10 m altitude in a sub-Antarctic region [14]. Here we tested UAV flights no less than 20 m height over the incubating plover. We conducted stepwise flights from 100 m down to 20 m with a 10 m interval, staying at each height for 1 min and observing the response of the incubating plover. We did not notice any significant behavioral responses of the plover during the flights. To obtain the common ringed plover images, we flew the drone at 20 m AGL for 5 min; the operator was in a tent 100 m away from the birds. The flight height for the nesting birds was lower (20 m) than that for the pink-footed geese (110 m) in the open water. After flight, we selected 5 high-quality clear and not blurred RGB images with sufficient overlap between images, as well as a single thermal image, as we did with the pink-footed geese images. Then, the RGB images were mosaicked in PhotoScan Pro, and the thermal image was co-registered to the mosaicked RGB image. The GSD of the mosaicked RGB images and coregistered thermal images were 0.95 cm and 3.68 cm, respectively. The thermal image was used to compare the plover temperature with the temperature of two surrounding areas (vegetation and rock, and stream). Using the ‘Create Random Points’ tool in ArcGIS 10.3 that we also used for geese thermal images, 100 random points were produced from the thermal image. Among the 100 points, 99 points were assigned to one of two surroundings (vegetation and rock, and stream), and 1 point was discarded because it was in a boundary. Additionally, three linear transect lines intersecting the nest and other surface types were drawn on the thermal image and were investigated by comparing the nest temperature with other environmental temperature profiles. For the blind survey, we operated UAV flights at 110 m height for 15 min and in a military restricted area with mudflats (approximately 0.05 km^2^) in Incheon, Korea; the operator was on a hill about 400m away from the birds. In consultation with the military office, we acquired permission to perform UAV flights for bird monitoring only and the operator stayed out of the restricted zone. We aimed to conduct a blind UAV flight test to monitor birds in the restricted zone with no previous knowledge. Similar to the geese monitoring, we determined the flight distance to over 100 m in height to avoid possible disturbances. A total of 138 RGB images were acquired along the muddy flat area (S1 Fig) and at least 9 images were overlapped in the mudflat area of 0.05 km^2^ (S2 Fig).

We did not observe any behavioral reactions, such as an alerted reaction with flying away or making alarm calls, during the drone flight. Initially, we did not know the species and number of birds existing in the mudflats. In the control screen, we observed white objects, so we took RGB and thermal images around the objects, such that images overlapped in a similar way as the images in the proceeding two cases (S3 Fig).

The air temperature and humidity at the time of the UAV flights were applied to the thermal analysis using FLIR Tools software (FLIR Systems Inc., Wilsonville, Oregon, United States). The birds and the surrounding areas were measured using temperature contours extracted from the selected and coregistered thermal images with a one degree interval. The temperature contours were delineated as lines that connect locations of equal temperature using ‘Contour’ tool in ArcGIS 10.3. The highest contour temperature values that were able to delineate and separate individual birds among the contours were designated as thresholds for delineating boundaries of bird pixels (e.g., [27]). The temperatures between the birds and other surface types were compared using the selected contours confining only bird pixels and a random sampling approach for other surface types overall the entire image. The longest length of the convex hull of each selected contour was designated as the conservative size of individual birds leading to exclusion of the pixels mixed with the surroundings. The temperatures were expressed with box-whisker plots; a median line separated the lower quartile from the upper-quartile boxes, extended from the lowest to the highest value.

The thermal images of the geese were visually investigated by three people, independently, and consistent identifications and counts were carried out and confirmed by the temperature contours. In the case of the plover, none of the three investigators was able to detect the plover using RGB images. Although the plover was represented as a few pixels in the thermal image, the distinctive thermal contrast between the plover and relatively cold backgrounds enabled the detection of the incubating plover by all three investigators. In the blind survey, all three investigators reported identical locations and counts of black-faced spoonbills using mosaicked RGB images, and then counts were confirmed by the thermal image.

## Results

### Monitoring of molting geese flocks

From one UAV flight, a total of 37 birds in three flocks (16, 16, and 5 individuals) were observed, and this result was consistent with the numbers counted by binoculars and portable zoom cameras by two regular researchers in the field. No significant reactions were observed during the UAV flight.

**Fig 2:**
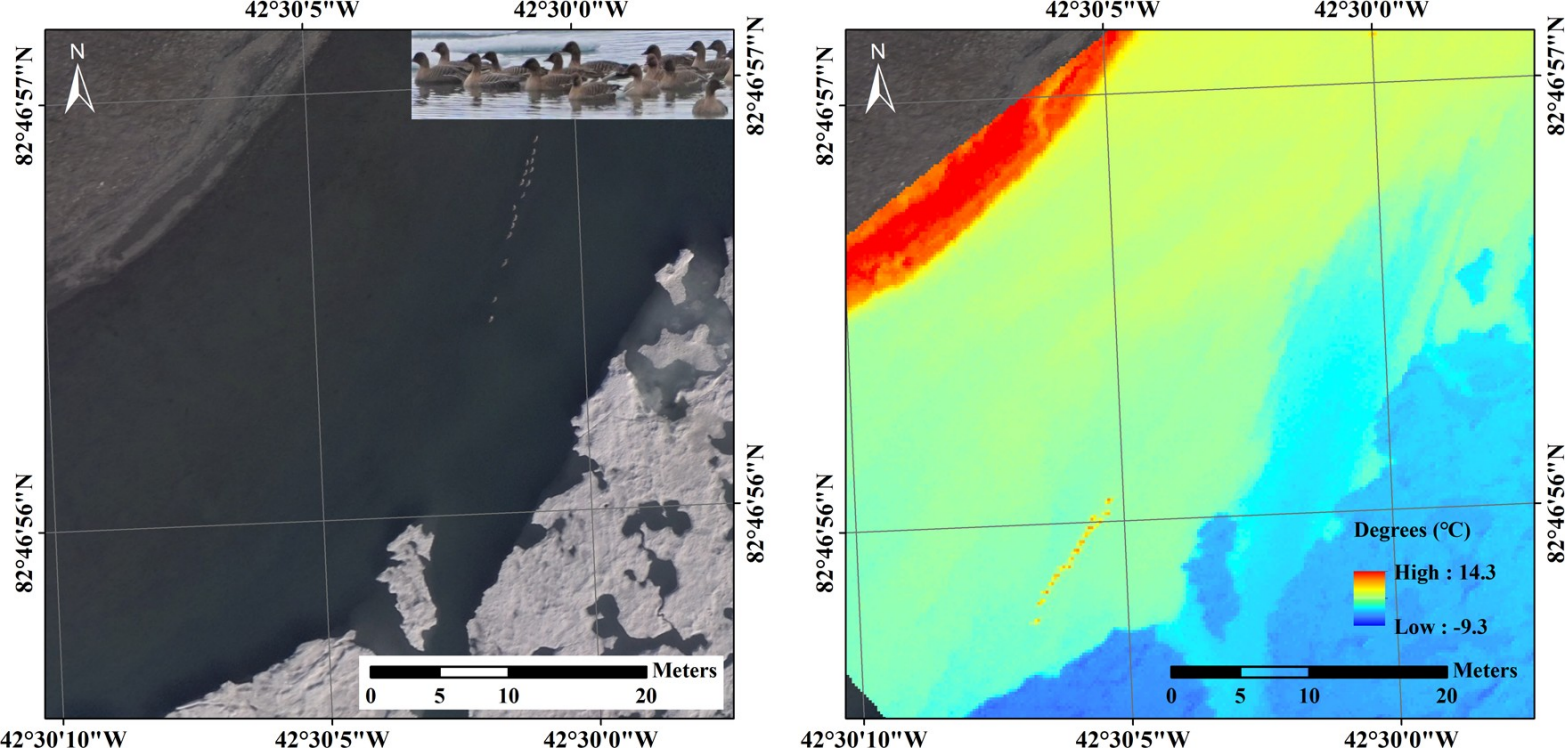
**Mosaicked RGB images (left, number of pixels = 69) and thermal images (right number of pixels = 24), which cover the geese and the surrounding area with sufficient overlap between images.** The pink-footed geese on the open water that were detected by binoculars by the researchers are presented (upper right in the left RGB image).

**Fig 3:**
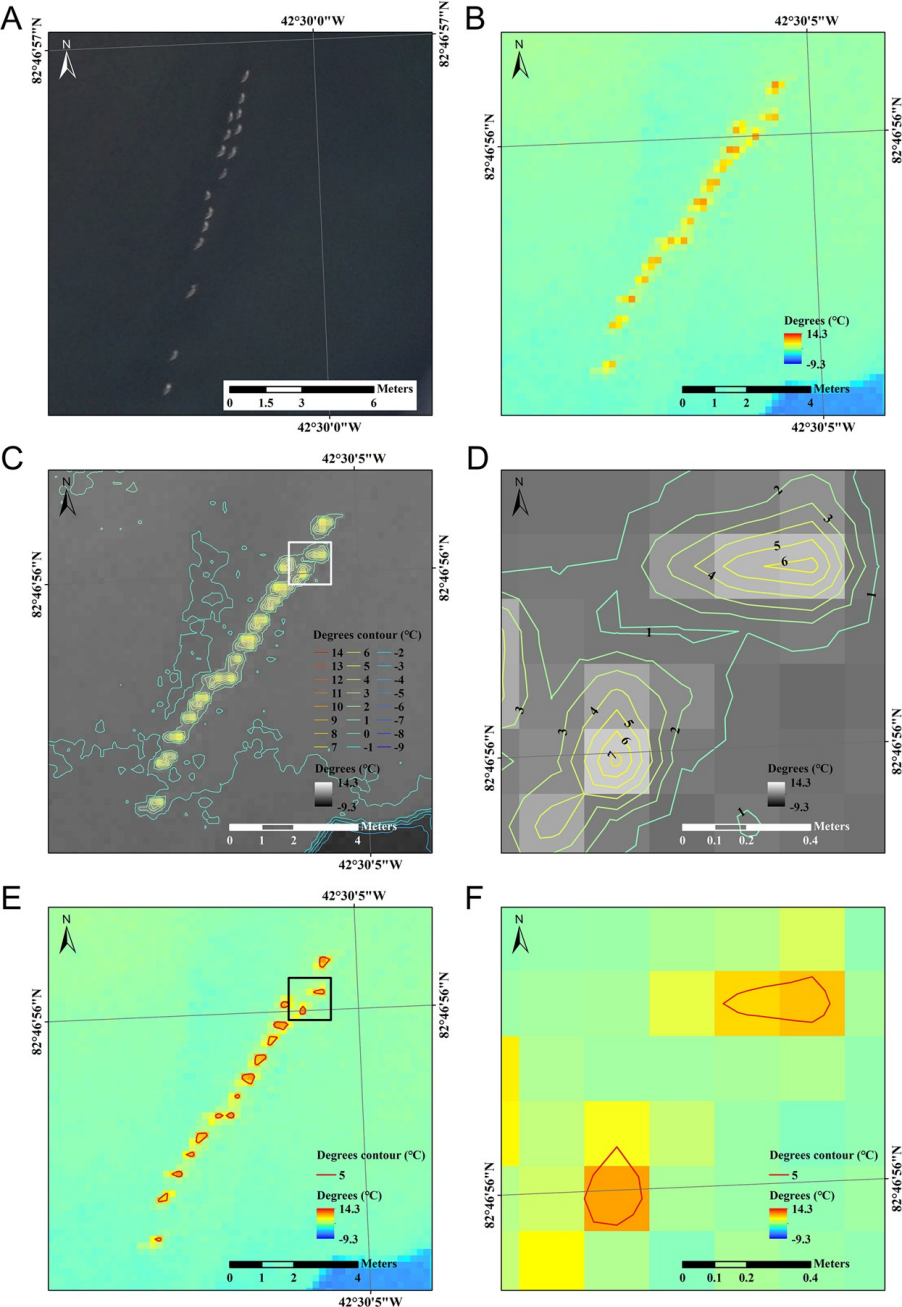
UAV images of pink-footed geese at the seashore. (A) Zoomed RGB mosaic image. (B) Zoomed thermal image. (C) 1°C-interval temperature contours in the thermal image. (D) Detailed view of the temperature contours around geese pixels within the area of the white box in (C). (E) Contours using a 5°C temperature threshold for a flock of sixteen geese. (F) Detailed view of the temperature contours with 5°C temperature threshold of the black box in (E) that provided the conservative proximate size of the geese (35 (± 2.5, SE) cm long).

In the mosaicked RGB images shown in Figs 2 and 3A, gray-colored round shape objects were observed and these were identified by the researchers using binoculars and potable zoom cameras (Fig 2, upper right in the left RGB image). The RGB images were manually taken by a remote control when birds were observed in the control screen and the thermal camera was automatically set to take images every second as stated above. Although the thermal image was coregistered to the mosaicked RGB image, the different strategies for taking images and selecting nonblurred thermal images caused differences in imaging time such that the geese had a different arrangement after the small imaging time gap between the RGB image and thermal image in Fig 2. In the thermal images of geese, the highest radiation temperature from the central part of the bird shapes was 14.3°C and the lowest temperature of the sea ice in the same image was -9.3°C (Fig 3B). In the contour images with a 1°C interval, the geese and the adjacent temperatures approximately ranged from 1 to 7°C, and the neighboring individual birds were not discriminated under 5°C (Fig 3A–3D). The degree contours at the 5°C threshold indicated that the 5°C contours formed sixteen polygons (Fig 3E and 3F), and this number (n = 16) corresponded to the counts from the RGB image.

**Fig 4:**
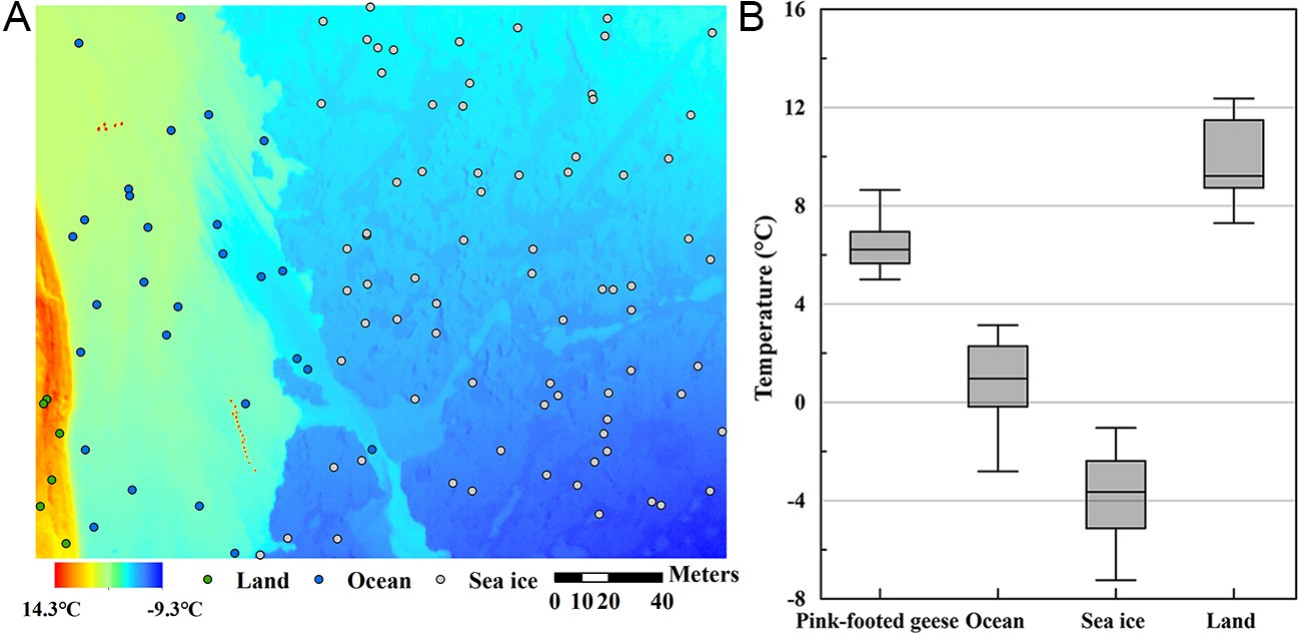
A thermal image including flocks of pink-footed geese and temperature distributions of each group of image pixels. (A) A UAV thermal shot at 110 m high of a goose flock (red dots), ocean (blue circles), sea ice (gray circles) and land (green circles). (B) Temperature distribution of 16 pink-footed geese body shapes and randomly selected dots in each group (ocean, sea ice and land) presented in box-whisker plots (a median line separates the lower-quartile from the upper-quartile, and whiskers extend from the lowest to the highest value) of the temperature distribution of the thermal image pixels in ocean, sea ice, land and pink-footed goose areas.

In the thermal image (Fig 4), two of the three flocks (16 and 5 individuals) were detected. During the image acquisition, one geese flock moved and was not included in the thermal image. The mean temperatures of the surrounding environments were 0.7°C (ocean), -3.8°C (sea ice) and 9.7°C (land), and the pink-footed geese had a temperature of 6.3°C (Fig 4). The random points assigned to the abovementioned environments were represented by 28, 76, and 6 pixels, respectively, with random points assigned to 84 pixels of pink-footed geese in the images (Fig 4). The RGB image and the selected 5°C contour in thermal images of a flock of geese provided the number of geese in the flock, sixteen, and the conservative and proximate size of the geese, which was calculated to be approximately 35 cm (± 2.5, SE) long. Although we tried to confine pixels representing geese only by temperature contouring, some edge pixels were contaminated with thermal radiation from both the backgrounds and the geese. To consider this mixing effect, the extracted sixteen geese polygons were adjusted to ±1°C contour polygons. The mean size of geese ranged from 19.1 to 50.6 cm for the ±1°C contour polygons. In the case of -1°C adjusted contour polygons (i.e., 4°C contour polygons), two pairs of geese polygons were merged into one polygon, and these merged polygons were not considered in the calculation of the size of the geese. In the case of +1°C adjusted contour polygons (i.e., 6°C contour polygons), one polygon was removed, as the selected 5°C was the highest temperature depicting an individual goose.

### Monitoring of an incubating plover

One UAV flight was conducted to record an incubating plover. No behavioral response was observed during the UAV flight.

**Fig 5:**
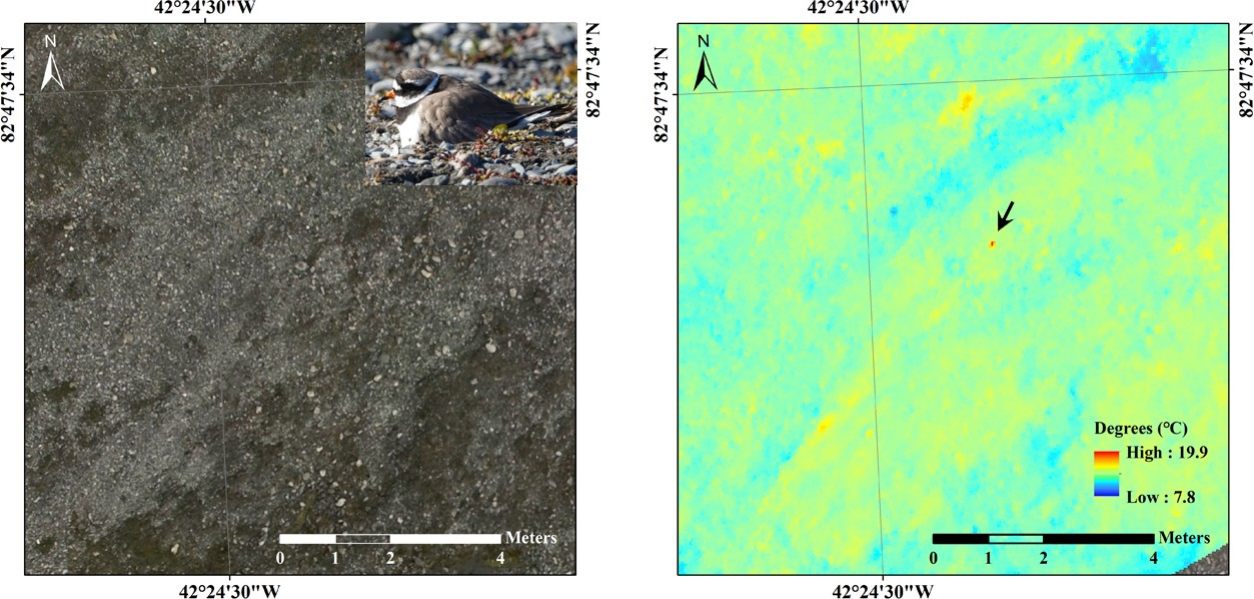
**A mosaicked RGB image (left) and a coregistered thermal image (right), which cover one incubating nest (indicated with an arrow).** The incubating plover that had been previously detected by the researchers during the survey is presented (upper right in the left RGB image).

**Fig 6:**
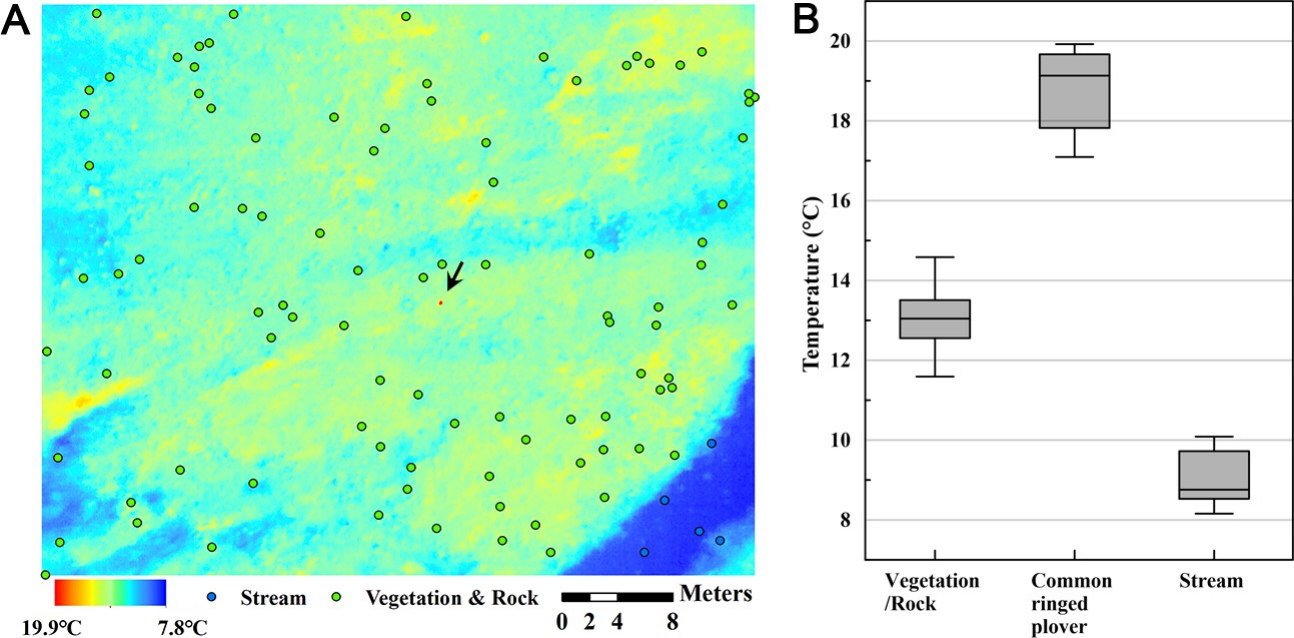
A thermal image shot including an incubating plover nest (indicated with an arrow) and the temperature distributions of each image pixel group. (A) A 20-m-high UAV thermal shot of an incubating plover (red dot), stream (blue circles) and vegetation and rock (green circles). (B) Temperature distribution of one common ringed plover body shape and randomly selected dots in each group.

**Fig 7:**
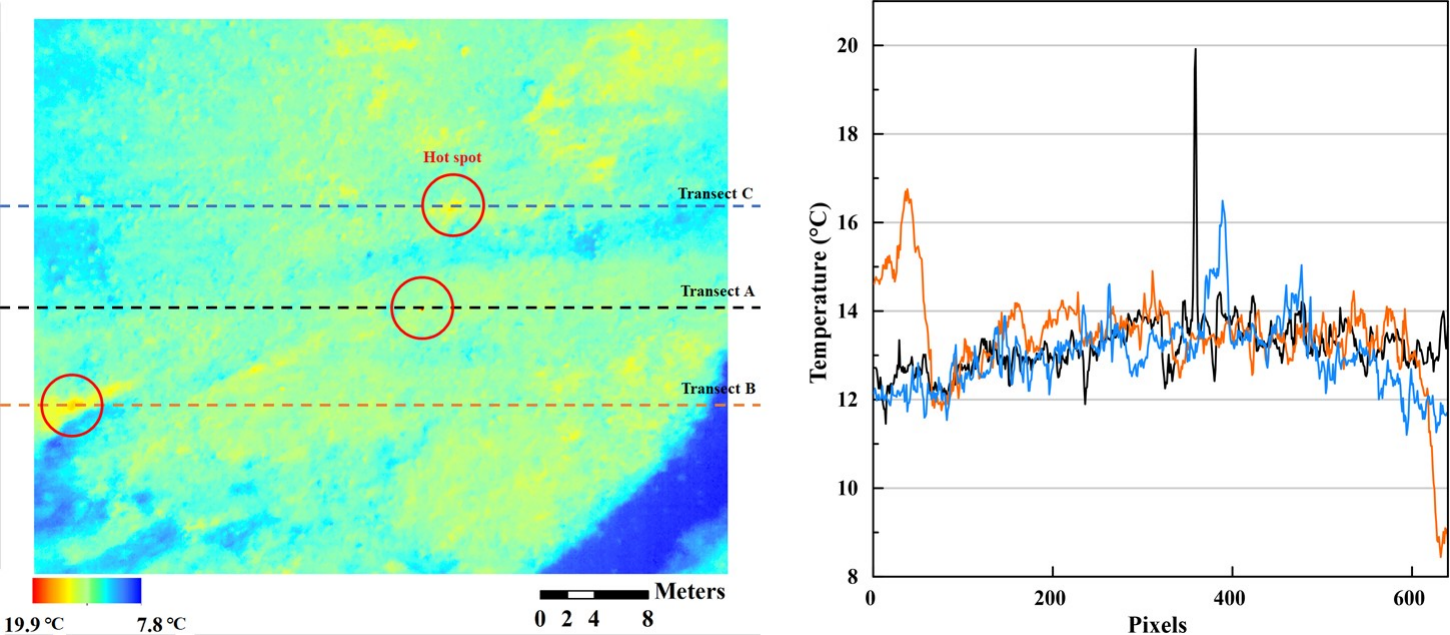
A thermal image near the plover nest and the surroundings (left) with three transect lines (A in black, B in orange, C in blue; three lines with hot spots) and the transect temperature profiles, one of which includes the incubating plover (Transect A; the pixel in the middle of the bird body was 19.9°C, and parallel lines ranged from 12 to 14°C).

The plover was not visible in the RGB image (Fig 5, left) but the thermal image visualized a candidate location for the nest, which had been previously found by a researcher, with a temperature up to 19.9°C. The temperatures of the incubating plover were distinctively higher than those of the surrounding environment (Figs 5 (right) and 6A). The mean temperatures of random points of the environments and the common ringed plover were 9.1°C (stream), 13.0°C (vegetation and rock) and 18.9°C (the common ringed plover), and each covered 5, 94, and 7 pixels, respectively (Fig 6). The plover temperature ranged from 17.1 to 19.9°C, and other surrounding temperatures ranged lower than those of plovers (9.8–14.6°C, vegetation and rock; 8.2–10.1°C, stream). The highest temperature among the thermal pixels was 19.9°C in the middle of the bird body, and this temperature was at least 5.6°C higher than the transect line (Transect A) temperature profile of other land surface types, which ranged from approximately 11.5–14.3°C (Fig 7). Although two additional transect lines (Transect B and C) passing through other hot spots were analyzed, the transect line with the common ringed plover pixels yielded a prominently higher temperature gap between the peak value and the other values (Transect A, 5.6°C) than the other two transect lines (Transect B, 1.6°C; Transect C, 1.7°C).

### Monitoring of birds in a muddy tidal area

**Fig 8:**
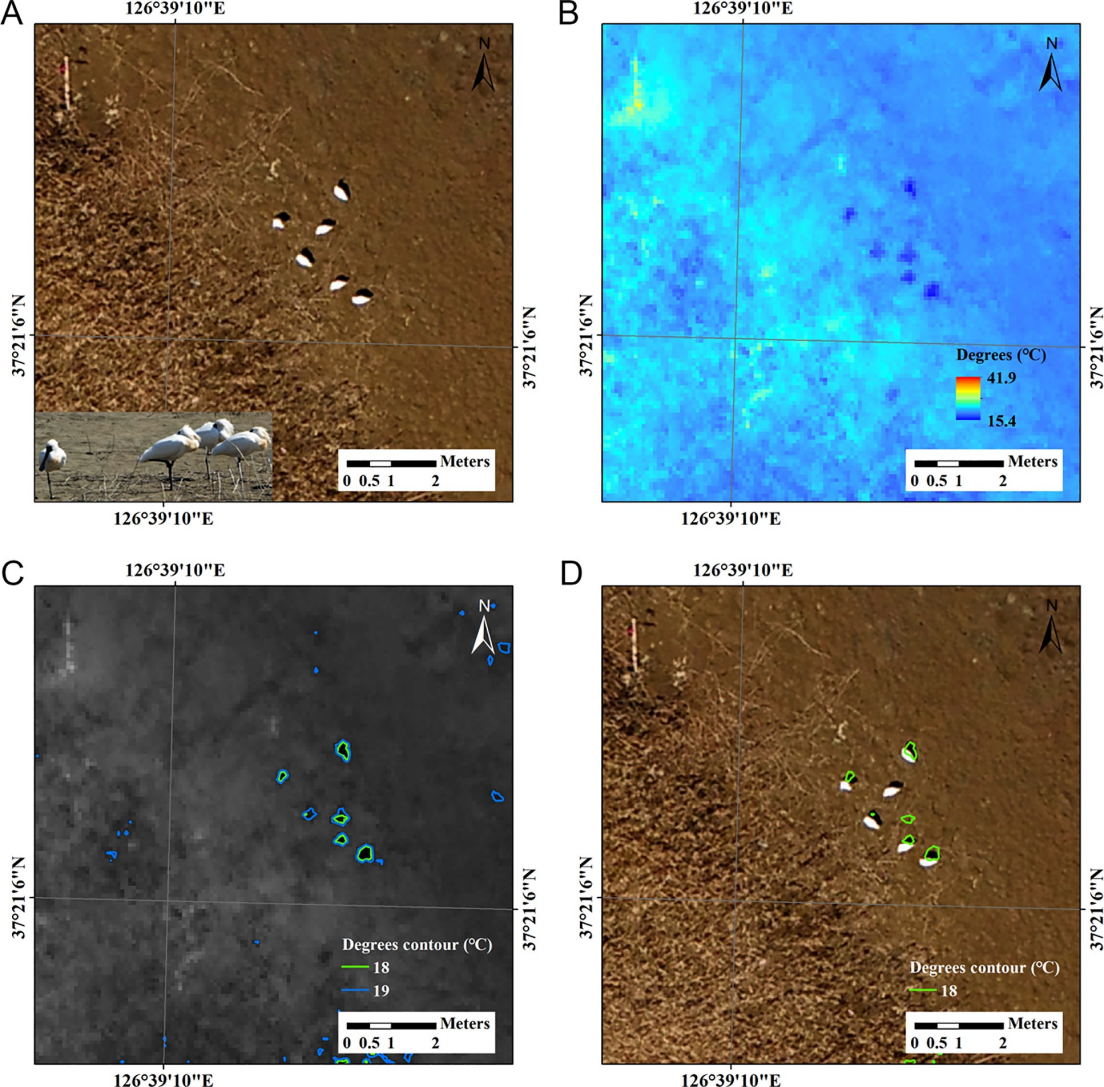
A blind survey with UAV images of white objects in a mud flat area. (A) Zoomed RGB mosaicked image at 110 m height. (B) Coregistered thermal image at the same spot with the RGB image. (C) Selected contours using an 18°C temperature threshold on a flock of six spoonbills provided countable round shape figures. (D) Selected temperature overlay on the zoomed RGB mosaicked image.

We conducted two separate UAV flights on the same spot and found no behavioral responses of the birds. Along the tidal line, we controlled the UAV and took 138 RGB images at one second interval (S1 Fig). With overlapping images, we could build a mosaicked RGB image (S2 Fig). Six white objects were observed in a small flock in the mosaicked RGB image (Fig 8A) and were also detected in a coregistered thermal image (Fig 8B and 8C). From the RGB and thermal image, we acquired the GPS coordinates (S1 and S3 Figs). At the same spot, a flock of black-faced spoonbills was observed by binoculars. By taking photographs of the objects, we could confirm the identification of the birds (Fig 8A, bottom left). In the thermal image, which was taken by the separate drone flight in 10 min after the RGB photo, the spoonbills had the lowest radiation temperatures (15.4°C) among the surroundings (Fig 8B). The mudflat temperature ranged up to 41.9°C under sunny weather. The contour images indicated that an 18°C temperature threshold provided countable round shape figures (n = 6) for a flock of spoonbills (Fig 8C). When the thermal image was overlaid on the RGB image, five objects were located on the shadow of the spoonbills and one object in the middle was approximately 50 cm away from the bird (Fig 8D).

## Discussion

Our thermal image results suggest that piloting UAV equipped with RGB and thermal cameras can be supplementary to conventional ground bird monitoring. In three case studies, we tested the effectiveness of a UAV combined with a thermal camera in an Arctic environment and in a restricted area. The study of the two arctic bird species showed that the numbers and the locations of the birds could be detected with the hotspots of thermal images. In contrast, the radiation temperatures of spoonbills were lower than those of the mudflat; nonetheless, their approximate numbers and locations could still be estimated with thermal images.

Our geese monitoring results suggest that UAVs may be confluent with traditional monitoring to detect flocks of 10–20 individuals. The thermal images and RGB images were sufficient to determine the round shapes of geese and count their numbers. Considering the typical goose body temperature (approximately 40–42°C of the internal body temperature), the radiation temperature of the pink-footed geese was quite low. This may be because the geese were covered with sea water while swimming and partially because of the mixing effect between the pink-footed geese and the sea water around the geese resulting from the large pixel size (20.37 cm). Additionally, animals are rarely observed to maintain the same temperature on their surface and in their core in order not to minimize heat loss [28]. Given the Arctic conditions, it is likely that the geese conserved their heat even during molting. We assumed that the uncertainty range of the temperature in a single thermal image is generally smaller than the image to image uncertainty (± 5°C) due to fundamental factory calibration of the detector array; thus, temperature differences smaller than ± 5°C could be discernable in the thermal image. However, the radiation temperatures (approximately 5–9°C) did not overlap with the sea water temperature (approximately 1–2°C), so the geese were distinguishable. When we set the threshold at 5°C, the geese individual temperature contours did not overlap each other (Fig 3D). The number of geese counted in the thermal image corresponded to the number counted in the RGB image. Thermal cameras can provide images to confirm the presence of the birds using a threshold temperature to separate birds from the surroundings.

For the common ringed plover monitoring, the UAV thermal camera provided 19.9°C spots in a 12–14°C transect line. The birds were not easily visible due to the small body size of plovers, but the temperature pixels of the bird’s radiation were higher than those of rocks and vegetation areas. However, we did not exclude the possibility that rocks were warmed enough that their radiation temperature was near the bird radiation temperature. Our study was conducted in clear sunny weather in the afternoon, so the vegetation and rock area showed a mean temperature of 13.0°C, which was only a 5.9°C different from the mean temperature of the plovers (mean temperature of the common ringed plover was 18.9°C). The plover temperature region might be caused by the combined effect of convex topographic features and land surface composition, which was mainly rocks. Limitation in the use of thermal images can occur when the temperatures of birds and other land surface types are similar to the temperatures of plover and other hot spots on land over diurnal or seasonal land surface temperature variation. The climatological study of land surface temperature (e.g., [29]) before conducting fieldwork can help high-contrast thermal images of high contrast be obtained. Thermal image acquisition under optimal timing with consideration of the thermal inertia, that is, the land surface’s capacity to conduct and store heat and radiate it outward [30], of each land cover type can result in enhanced thermal contrast among various land covers in acquired images.

A flock of black-faced spoonbills was detected in a blind survey in the military restricted zone. After taking UAV images, we confirmed the birds by binoculars and portable cameras at rising tide when the birds walked to a visible place outside of the restricted area. Contrary to our expectation, the radiation temperatures of spoonbills (approximately 15–19°C) were lower than those of the mud flat area (up to 41.9°C). In our results, the threshold temperature in the contour image was set to 18°C to detect the individual birds. One possibility is that the white body of the birds could increase the reflectance of light, resulting in a lower temperature than the temperature of the surroundings. Another possibility is that the thermal data could reflect the cool spots produced by the shadows of the birds. When RGB and thermal data were overlapped with 10 min time interval, five thermal shape of 18°C contour were located in the shadows of the birds and one thermal shape was located about 50 cm away from the bird. Although there were no particular reactions detected in the sky with the binoculars, this may be due to the slight movement of the mismatched bird, which possibly move during the two drone flights so that the thermal image was in the place where the bird had originally stood in the first flight. The dark spots which look like thermal shadows have different shapes to the birds in the RGB images and in fact look more like the shadows that the birds are casting in the RGB images.

Black-faced spoonbills are ranked as Endangered (EN) in the IUCN Red List, and the total number of adult individuals is expected to be 2,250. Korea is one of their breeding sites. Thus it is very important to monitor Black-faced spoonbills near the breeding sites. UAVs are highlighted as conservational tools to detect protected species or to surveil their external conditions. Sumatran orang-utans and Sumatran elephants were detected and counted by conservation drone [17, 19]. Similarly, Mulero-Pázmány et al. [18] tested a rhinoceros anti-poaching system including fixed-wing aircraft. To protect the Western capercaillies (*Tetrao urogallus*), Weber and Knaus [31] investigated human disturbance such as snowshoe tracks with camera-mounted fixed-wing aircraft in the Entlebuch UNESCO Biosphere Reserve. GPS data loggers were attached to a couple of lesser kestrels (*Falco naumanni*), and their track was imitated by UAV to understand their landscape use through mosaicked images [32].

As a pilot study to test a UAV-thermal camera system for the detection of bird species, we evaluated thermal images captured using a UAV for efficacy in counting birds on sea ice and in detecting cryptic bird nests. A single flight of 20 min was enough to cover three flocks 500 meters away from the seashore, and a flight of less than 5 min distinguished one candidate spot for the nest from the surroundings in 10 square meters that we had previously checked during the survey. The UAV images also provided approximate body size, but the bird lengths from above may not reflect actual bird sizes because the images show 2D projections of 3D objects. Nevertheless, in a habitat with low species diversity, it can be useful to distinguish the birds roughly by body length.

In future studies, UAV thermal systems can be used to monitor seabirds in harsh environments and human-restricted areas where researchers are not easily able to detect wader nests from highly cryptic backgrounds. However, we do not assume that this method can be a replacement of conventional surveying methods. Instead, we think that this method can provide complementary data to distinguish living creatures from environments. For detecting bird nests during incubation, thermal images can be used to search for possible nest candidates before humans perform a field survey. Also, this study based on the interpretation by researchers with photo images that RGB and thermal cameras acquired. This method lacks processing images to classify the patterns of the images and identify animals with the distinctive species-specific characteristics such as their shapes and sizes. Thus, the image processing approaches would improve efficiency and accuracy for animal counting and identifying with UAV imagery.

We consider the ethical issues of approaching birds using UAVs. A few studies have examined the behavioral responses of wild animals to approaching devices [12, 26, 33] and, can provide relevant guidelines. Among these studies, Vas et al. [26] used a small quadcopter, which was similar to our machine, to approach mallards, greenshanks, and flamingos. Although the authors did not find any measurable impacts within 4 m distances, they suggested avoiding vertical approaches and launching the drone more than 100 m from the animals. The geese and spoonbills might have been alerted to the approaching drones but we did not notice any significant responses in the drone images of 110 m flights. Additionally, we found no particular reactions from the incubating common ringed plover. The plover did not show any behavioral responses to the UAV at a height of 20 m. Because the plover was in the middle of the incubation period, the bird may remain still even if disturbed. However, the geese were in the molting period and seemed to be very sensitive to any moving objects. Even if the flight height was greater than 100 m and higher than previous airplane approaches [22], we do not exclude the possibility that the molting geese were affected by the UAV approaches. Thus, we think that it may be necessary to estimate safe approach guidelines by conducting careful flight approaches for different bird species considering breeding characteristics.

## Supporting information

S1 Fig Results of image mosaicking using 138 RGB images. (A) Image locations and mosaicked images. Red dots indicate the exact locations of RGB image acquisition during the UAV flight. In the black square, white dots were detected. (B) A zoomed image around the white dots which were suspected to be birds.
(TIF)

S2 Fig Number of image overlaps of the RGB images acquired during the blind survey. (TIF)

S3 Fig A thermal image overlaid on the mosaicked RGB image for a blind survey. (TIF)
