# Supplementary figures and images for: Detection of two Arctic birds in Greenland and an endangered bird in Korea using RGB and thermal cameras with an unmanned aerial vehicle (UAV)
Source: PLoS One. 2019 Sep 4;14(9):e0222088. doi: 10.1371/journal.pone.0222088 (PMC6726231; doi:10.1371/journal.pone.0222088)

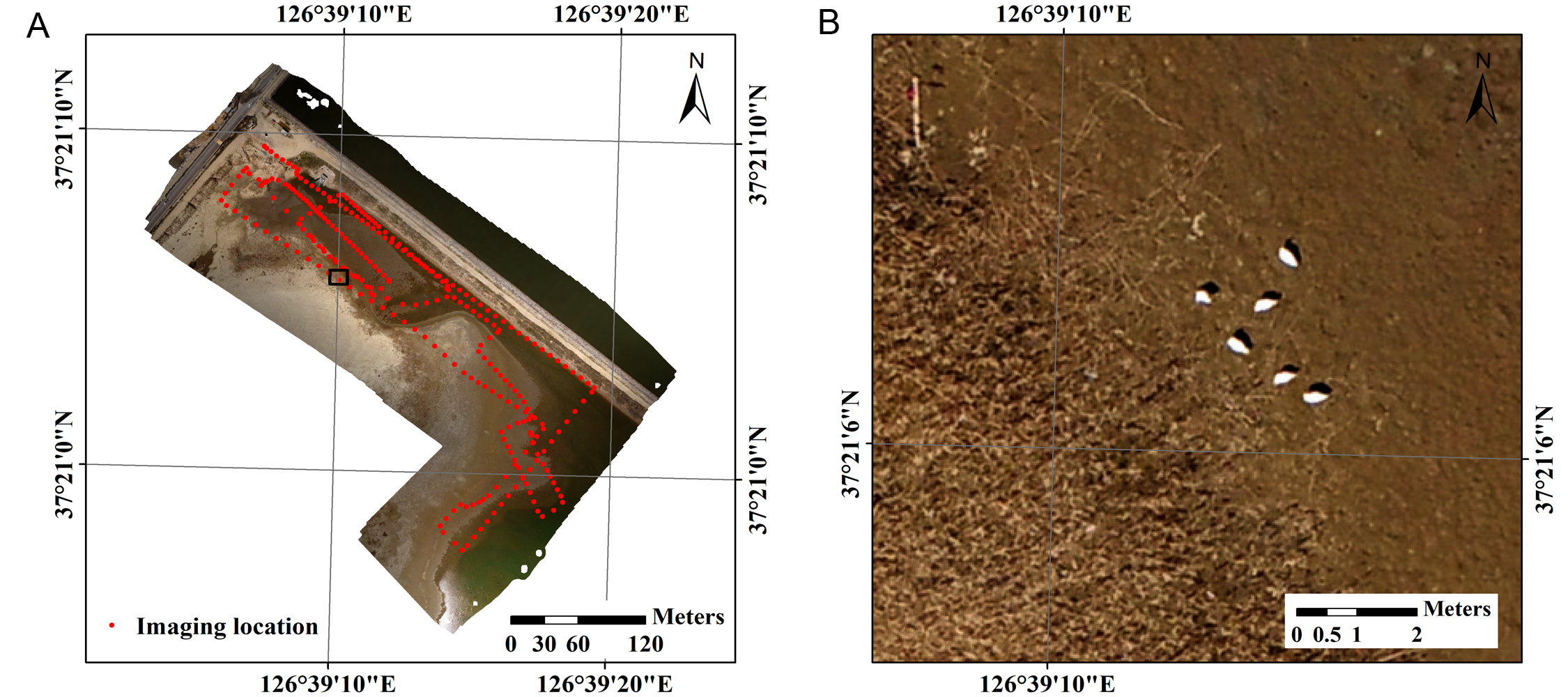

Supplement: S1 Fig — (A) Image locations and mosaicked images. Red dots indicate the exact locations of RGB image acquisition during the UAV flight. In the black square, white dots were detected. (B) A zoomed image around the white dots which were suspected to be birds. (TIF) [file pone.0222088.s001.tif]

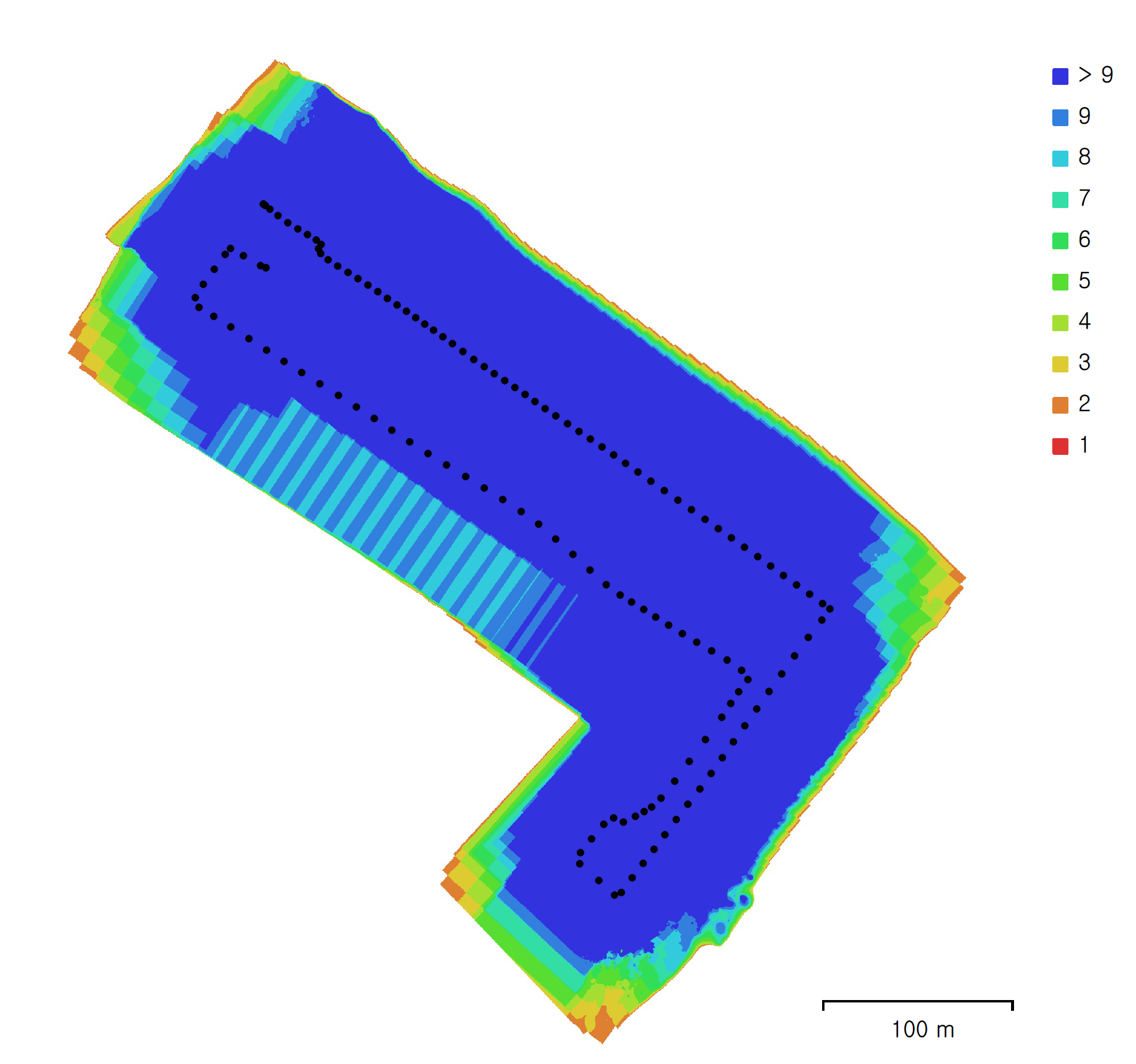

Supplement: S2 Fig — (TIF) [file pone.0222088.s002.tif]

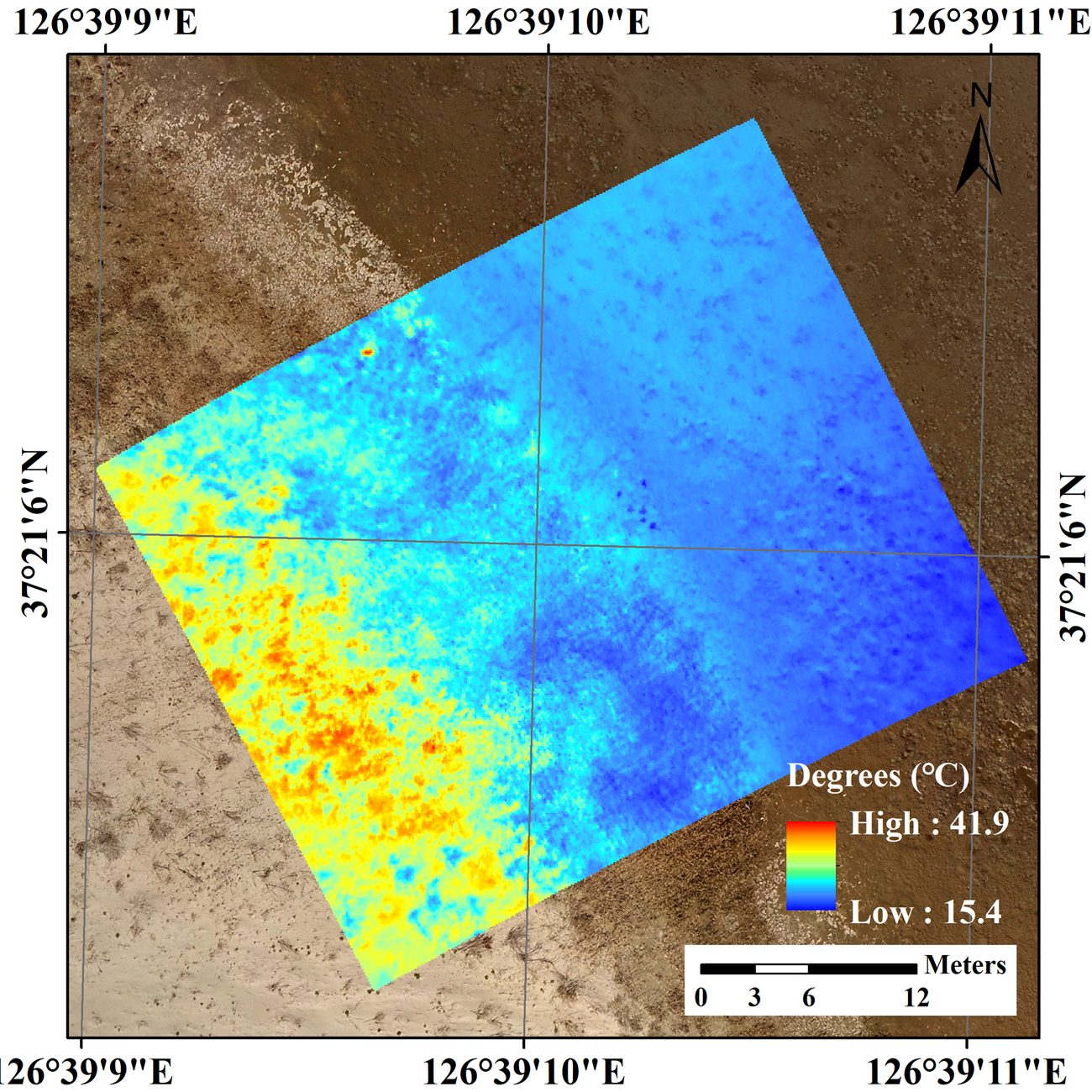

Supplement: S3 Fig — (TIF) [file pone.0222088.s003.tif]
